# Opportunities and Challenges of Multiomics for Discovery and Monitoring of Human Pathogens

**DOI:** 10.1021/envhealth.5c00040

**Published:** 2025-07-09

**Authors:** Zoe Turner, Andrei P. Drabovich

**Affiliations:** Division of Analytical and Environmental Toxicology, Department of Laboratory Medicine and Pathology, Faculty of Medicine and Dentistry, 3158 University of Alberta, Edmonton, Alberta T6G 2G3, Canada

**Keywords:** multiomics, meta-omics, meta-genomics, meta-proteomics, next-generation sequencing, mass spectrometry, pathogens, environmental monitoring, clinical diagnostics, human health

## Abstract

Detection and monitoring of pathogens is a central aspect of maintaining public health. Rare and neglected zoonotic viruses have the potential to evolve and expand exponentially, leading to unforeseen outbreaks, epidemics, and pandemics. The emerging multiomics and meta-omics techniques and workflows, such as proteogenomics and meta-genomics, offer the potential for the detection of harmful pathogens, as well as opportunities for the discovery of previously unknown bacterial, parasitic, or viral pathogens. Multiomics and meta-omics workflows provide molecular information for tracking pathogens and understanding the effectiveness of spread mitigation strategies. In addition to environmental monitoring, multiomics and meta-omics approaches have the potential for clinical applications and in-depth characterization of novel pathogens. In this review, we discuss recent applications of multiomics and meta-omics techniques, their advantages over traditional methods, and their potential implementations in biomedical research, environmental studies, and healthcare. We critically assess the benefits and challenges of multiomics and meta-omics studies and discuss their future perspectives.

## Graphical abstract

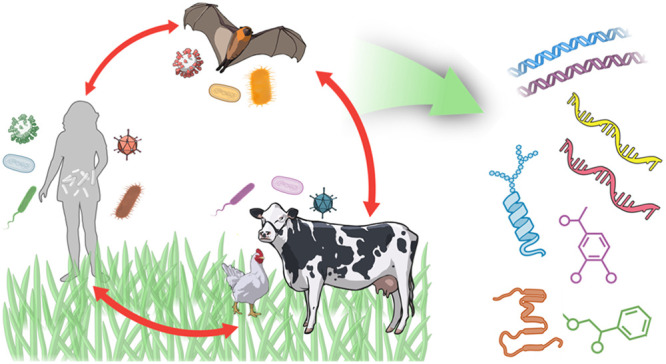

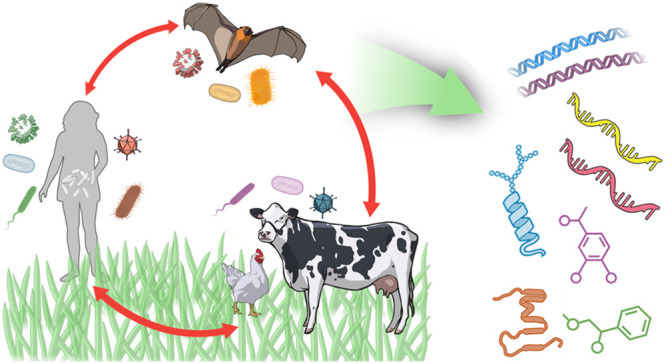

## 1 Introduction

The invisible world of microorganisms, including bacteria, fungi, and viruses, can be found in every environment, spanning from damp tropical forests to frigid polar deserts, hot acidic lakes, extreme pressure oceanic trenches, and potentially extra-terrestrial environments.^1^ Microorganisms vastly outnumber any other organisms in the ocean, soil, plants, and animals.^2^ While some microorganisms have evolved to have strictly symbiotic relationships with the environment and humans, the potential for random mutagenesis and exponential growth could rapidly shift the balance and turn symbiotic microorganisms into deadly infectious strains, with the pathogenic *Escherichia coli* strain outbreaks being the prominent examples.^3^ Thus, understanding microbial evolution and the determinants of environmental and human health can potentially prevent and mitigate disease outbreaks. Pandemic preparedness with a focus on the detection and investigation of potentially harmful and pathogenic microorganisms is a recent shift in public health initiatives and scientific research. Over the recent decades, an increase in emerging infectious diseases has been reported, with the majority of diseases (∼60%) originating and crossing from animals.^4^ A 2008 meta-analysis revealed that the emerging infectious diseases were mostly caused by bacteria or rickettsia (54%), typically from drug-resistant strains, followed by viral pathogens (25%).^4^ However, new human viruses are being discovered continuously, with a steeper increase in viral species discovered over the last two decades due to introduction of multiplex and high-throughput analytical assays, such as next-generation sequencing methods. Since 1980, newly discovered pathogens have been disproportionately viral, and by 2007, 189 human viruses were known.^5^ From 2012 to 2022, the number of known human viruses had risen from 219 to 270.^6,7^ The continuously growing number of known human viruses emphasizes the vast amounts of unexplored viromes. In the past two decades, there have been six major outbreaks due to viral agents (Table 1). Environmental monitoring and discovery of novel viruses with the potential to infect humans are at the forefront of pandemic preparedness.

**Table 1:** Major Viral Outbreaks, Epidemics, and Pandemics in the 21st Century with Death Toll Greater than 500^a^

year	event	virus	death toll	source
**Outbreaks**				
2002	SARS outbreak	SARS-CoV-1	774	^10^
2009	West African meningitis outbreak	meningitis	2449	^11^
2010, 2019	measles outbreaks in Congo	measles	11,518+	^12,13^
2012	MERS outbreak	MERS-CoV	940	^14^
2015	Indian swine flu outbreak	influenza A H1N1	981	^15^
2017	Gorakhpur hospital deaths	Japanese encephalitis	1317	^16^
2024	HMPV outbreak in East Asia	metapneumovirus	unknown	^17^
**Epidemics**				
2004	Indonesia dengue epidemic	dengue	658	^18^
2006	Philippines dengue epidemic	dengue	1017	^19^
2008	China hand, foot, and mouth disease	enteroviruses	3322+	^20^
2013	Western African Ebola virus epidemic	Ebola	11,323+	^21^
2013	avian influenza epidemic	influenza A H7N9	511	^22^
2018	Kivu Ebola epidemic	Ebola	2299	^23^
2019	dengue fever epidemic	dengue	1206	^24^
2023	African mpox epidemic	Mpox	812	^25^
2024	American dengue epidemic	dengue	7700	^26^
**Pandemics**				
1981–present	HIV/AIDS pandemic	HIV	42 million	^27^
2009–2010	swine flu pandemic	influenza A H1N1	284,000	^28,29^
2019-present	COVID-19 pandemic	SARS-CoV-2	7–36 million	^28,30^

a According to the World Health Organization, an outbreak is the occurrence of disease in excess of normal expectancy. An epidemic is the rapid spread of disease to a large number of individuals in a given population within a short period of time. A pandemic is a disease that has a sudden increase in cases, a substantial number of cases, and rapid spread across large regions, continents, or worldwide.

While microbiome research has rapidly advanced within the past decade allowing for the characterization of diverse microbial communities, research on the virome, named the “dark matter of biology”, is still lagging as vast numbers of viruses are still yet to be discovered and characterized.^8^ One limiting aspect of virome studies is the reliance on culture-dependent methods for pathogen discovery, since viruses require a host for replication. Culture-independent methods are being developed and improved to facilitate deep virome profiling and tracking through meta-genomic sequencing of the entirety of the viral genomic material in clinical and environmental samples.^9^ Emerging multiomics and meta-omics workflows provide novel and powerful tools to discover and monitor symbiotic microorganisms, and harmful pathogens present in the environment, animals, and humans.

Our review provides a broad overview of existing meta- and multiomics techniques, as well as their integration, from a pandemic preparedness perspective. We overview the importance of meta-omics for spread mitigation effectiveness testing, tracking viruses with the potential to cross from animals to humans, and exploring the potential use of meta-omics as novel tools in biomedical research, environmental studies, and healthcare. We re-emphasize the challenges of multiomics and meta-omics techniques and critically review the issues associated with meta-omics data analysis and interpretation, data integration, and unmet promises of multiomics health initiatives.

In this review, we define multiomics as integration of two or more -omics techniques and workflows across molecular types, such as DNA, RNA, proteins, and metabolites (Figure 1). Meta-omics is defined as integration of analysis across two or more species, such as hosts and pathogens. Unlike single omics (e.g., proteomics) and multiomics techniques (e.g., proteogenomics), meta-omics analysis targets the full molecular and species diversity of the sample, including contributions from the environment, media, microorganisms, and hosts.

**Figure 1:**
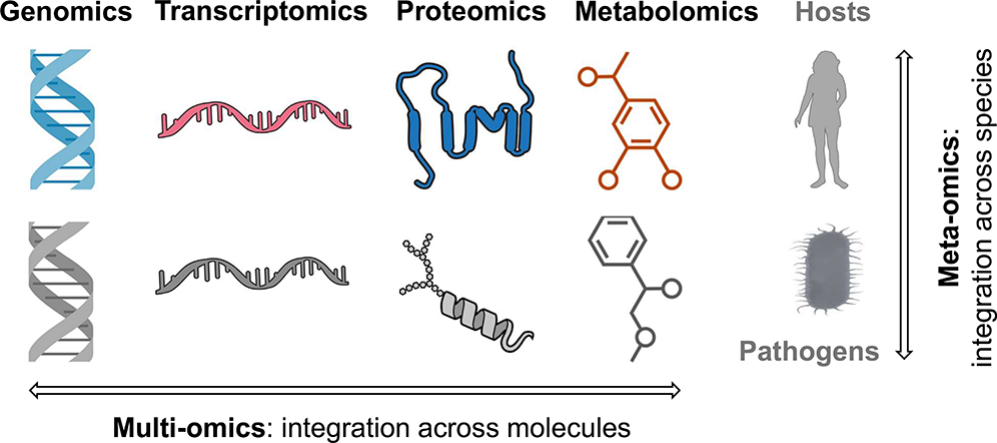
Comparison of multiomics and meta-omics workflows. Multiomics is defined as integration of two or more -omics techniques and workflows across molecular types, such as DNA, RNA, proteins, and metabolites. Meta-omics is defined as integration of analysis across two or more species, such as hosts and pathogens.

## 2 Conventional Methods to Detect Pathogens

Conventional methods for identification of pathogens include culture-based approaches followed by pathogen cell detection using microscopy, as well as pathogen-specific enzymatic assays and antigen immunoassays.^31^ Conventional methods, however, suffer from low selectivity and long turnaround time and are not practical for viral pathogens. Another limitation of conventional tests is their targeted approach focused on a known pathogen or groups of pathogens (for example, Gram-, catalase-, or coagulase-positive bacteria).^32^ Recently developed multiplex assays based on polymerase chain reaction (PCR) or reverse-transcriptase-PCR (RT-PCR) enable the simultaneous detection of several pathogens and are replacing the traditional single-analyte tests.^31^ Multiplex PCR tests, however, require thorough optimization of assay parameters (primer concentration, buffer concentration, cycling temperatures, etc.) before practical implementation in clinical and environmental studies.^33^ Since the majority of the virome species is not yet known, multiplex PCR or RT-PCR may result in false-negative identifications of emerging infectious diseases.^34,35^ Microarrays mostly detect known pathogens and may also identify unknown related species.^36,37^ It should be noted that the increasing number of tested pathogens increases the chance for cross-reactivity and false-positive identifications and may require independent validation with single-pathogen tests. Overall, conventional approaches and targeted methods provide straightforward answers regarding the presence of known pathogens but are less practical to detect novel pathogens.

## 3 Meta-Genomics

Meta-genomics is a powerful tool to identify emerging pathogens based on their nucleic acid sequences. Meta-genomics reveals the genomes of numerous bacteria, fungi, viruses, or parasites in complex environmental and clinical samples in an unbiased way.^31,32^ An overview of a conventional meta-genomic analysis is shown in Figure 2. Meta-genomic analysis can undergo two approaches: untargeted shotgun meta-genomic sequencing of all nucleic acids in the tested sample, including those of previously unknown pathogens, as well as targeted sequencing of known pathogens.^38^

**Figure 2:**
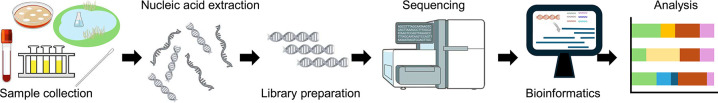
The NGS meta-genomic pipeline. Meta-genomic analysis starts with sample collection of clinical or environmental samples. Clinical samples such as urine, blood, tissue, or swabs may require depletion of host DNA to increase sensitivity of analysis of microbial genomes. Culturing clinical samples on agar also depletes host DNA while amplifying microbial genomes. Meta-genomic analysis of viral species may require extra filtration to remove nonviral components prior to DNA and RNA extraction. Similarly, environmental samples such as water or soil require isolation of bacterial cells and viral particles before cell lysis and nucleic acid extraction. For air samples, the biological particulates are extracted from air using filters. Library preparation typically includes amplification of DNA and cDNA (following reverse transcription of RNA into cDNA), DNA fragmentation and addition of sequencing adapters specific to each sequencing platform. Next-generation sequencing platforms enable massive parallel sequencing of nucleic acids, and bioinformatic approaches are used to assemble and align sequencing reads. Sequencing reads are either mapped against the reference genomes via database searching or *de novo* assembled into novel genomes.

*De novo* shotgun meta-genomics pipelines enable identification of unknown sequences and detection of previously known pathogens missed due to novel mutations at the primer binding sites.^39^ *De novo* sequencing involves assembling a complete genome from overlapping and continuous sequencing reads without relying on a reference genome. The process begins with initial assembly using algorithms such as SPAdes,^40^ Lasergene,^41^ or Flye^42,43^ to generate contiguous sequence blocks, or “contigs”, thereby mitigating errors through overlap alignment. A critical next step involves resolving genomic repeats, with long-read sequencing being a highly advantageous platform for this purpose. The draft assembly is then subjected to polishing aimed at correcting misassemblies, mismatches, and indels.^44,45^ Typically, this step consists of running the assembled genome through several algorithms, such as Arrow, FreeBayes, and Racon to identify and fix errors. Once polished, the assembled genome is compared against known sequence databases using tools such as UBLAST to annotate conserved elements and assess phylogenetic relatedness. As meta-genomics software rapidly evolves, new assembly and polishing tools are continuously emerging. For example, Latorre-Pérez et al. evaluated various assemblers and polishers using mock microbial communities and concluded that long-read data alone could be sufficient to generate accurate and complete genomes.^46^ Importantly, the choice of sequence assembler significantly impacted quality, particularly when software was designed specifically for meta-genomics (e.g., metaFlye, Raven, and Canu). Overall, the study concluded that while long-read sequencing alone may be adequate, software selection should be guided by specific study constraints such as time, computational resources, and data types. *De novo* approaches, however, require stringent and independent validation to claim the presence of novel genomes. Mistakes such as misalignments, mismatches, and indels cannot be avoided in meta-genomics sequencing but repeated polishing of assembled genomes and PCR confirmation enables confidence in the assembled genomes. It should be re-emphasized that *de novo* sequencing is statistically limited by the completeness and quality of raw data, which often results in missing or ambiguous sequences, and higher FPRs relative to database searches for known sequences. Unless *de novo* sequencing and *de novo* assembly are the only viable approaches (novel mutations, hypervariable regions of antibodies, novel microorganisms), searches of canonical curated database should be prioritized in data acquisition and analysis workflows.

Meta-genomics is an innovative tool with numerous applications in microbiology, public health, and medicine. The applications of meta-genomics are vast and rapidly growing. In this review, we explore only a few exemplary applications of meta-genomics in environmental and human health studies.

### 3.1 Overview of Meta-Genomics Platforms

Current meta-genomic studies are typically performed using next-generation sequencing (NGS) platforms provided by Illumina, Pacific Biosciences of California (PacBio), and Oxford Nanopore Technologies (ONT), each with advantages and limitations. Illumina is one of the most common NGS platforms due to its high throughput and lower costs per sample, even with some recognized limitations such as GC biases.^47,48^ Illumina NGS instruments utilize paired-end adapters for sequencing and have shown their utility over other methods in microbiome studies.^49^ Illumina sequencing enabled a more comprehensive coverage of the oral microbiome compared to phyloarrays or massively parallel pyrosequencing. A study evaluating three NGS platforms on a mock bacteriophage community demonstrated that the Illumina platform had the highest identification rate (11 of 15 genomes) with the fewest errors compared to PacBio or ONT alone.^48,50^ The combination of Illumina with PacBio or ONT sequencing substantially increased the number of complete genome assemblies (>97% coverage) and further lowered error rates.^48,50^ The PacBio and ONT platforms require careful preparation of sequencing libraries as the results could be skewed due to the length of input DNA.^48,51^ The advantages of PacBio include long-read *de novo* sequencing of genomes, as shown by multiple groups.^44^ PacBio enables direct sequencing of DNA and discovery of native epigenetic modifications.^52^ The ONT platform provides comparable sequence coverage as PacBio but is also portable, making it useful for field environmental studies and clinical point-of-care testing.^48^ ONT sequencing has dramatically improved its accuracy over the past decade. The latest upgrades in reagent chemistry, R10.4 flow cells, duplex mode sequencing, and improved base-calling algorithms result in an accuracy of Q40 (99.99%) to Q60 (99.9999%) for bacterial genome sequencing.^53,54^ In addition, R10.4 and RNA flow cells enable direct DNA and RNA sequencing, thus eliminating amplification-based errors and providing detection of native epigenetic modifications.^55^ However, direct sequencing requires further accuracy improvements, which currently stands at ∼88–90%.^56^ The choice of an NGS platform for meta-genomic studies is typically determined by a fit-for-purpose approach that considers the study objectives (either identification of known pathogens or *de novo* sequencing), complexity and diversity of microbial communities (long-read sequencing for highly complex and short-read sequencing for less complex samples), sample types (rapid short-read sequencing for clinical samples and slow long-read sequencing for complex environmental samples), and sample quality (short-read sequencing of degraded sequences in preserved clinical or wastewater samples, and long-read sequencing of intact nucleic acids isolated from bacterial cultures or unpreserved clinical and environmental samples).^57^ To summarize, different NGS platforms have their unique advantages tailored to specific applications in environmental and clinical studies, such as higher throughput, higher accuracy, *de novo* sequencing, in-field deployment, and point-of-care testing.

### 3.2 Environmental and Clinical Applications of Meta-Genomics

Shotgun meta-genomic analysis has been used in various contexts, such as environmental screening of microorganisms and microbiomes, and identification of bacterial and viral pathogens in clinical settings. Viral meta-genomic studies on marine sediments revealed the extensive viral diversity and dynamic range of the environmental microbiomes.^58^ The estimates revealed approximately 10^4^ viral genomes per kilogram of sediment, with a large fraction (75%) not related to any previously reported viruses and thus missed using targeted PCR measurements. Likewise, meta-genomic sequencing of marine water samples identified a large fraction of previously unknown viral genomes.^59^ The vast diversity of the discovered viral genomes was summarized as “···all the amphibians or reptiles known on the planet are less diverse than the viruses in only 1 kg of near-shore marine surface sediment”.^58^

Meta-genomics is an advantageous tool for environmental monitoring and may provide early warning of viruses with zoonotic potential, enabling outbreak preparedness and prevention. Zoonotic pathogens, animal pathogens that evolve to infect humans, are a growing concern due to increased animal–human interaction driven by environmental changes like deforestation.^60,61^ An estimated 60% of emerging human pathogens are of zoonotic origin, including wildlife and domesticated animals.^61^ Transmission may occur via direct contact with reservoir hosts, such as bats, or through intermediate hosts, such as pigs, chickens, horses, or primates.^62^ The direct meta-genomics analysis has been performed on reservoir animals such as bats to identify viruses with epidemic potential (Ebola, SARS, and rabies).^63–65^ As commonly observed in viral meta-genomic studies, the bat virome had low matches to known nucleic acid or protein sequences, demonstrating the extent of unknown or distantly related viruses currently residing in bats and indicating the vast potential of an unknown virus for cross-species transmission.^66,67^ The first bat *nairovirus*, as well as bat *rotavirus*, *gammaretrovirus*, and several new vertebrate, insect, and plant viruses were identified.^66^ In addition to the discovery of novel viral species, meta-genomics confirmed the circulation of MERS-related viruses and detected the spread and persistence of viral families within bats.^67^ While such monitoring strategies are currently not feasible for routine screens due to long turnaround time and high costs, further improvements in sequencing technologies and data analysis workflows will enable broader applications of meta-genomics for zoonotic reservoir monitoring.

Development of novel meta-genomic workflows to quantify the effectiveness of public health mitigation strategies is an emerging need. Reducing the number of viral particles in air, water, or soil is considered a key strategy to mitigate disease spread. Bioaerosols, the airborne particles originating from a biological source, are of particular concern in confined and insufficiently ventilated spaces, such as hospitals, schools, shopping malls, airports, and livestock shelters. Due to this risk, animal industries have implemented strict biosafety measures to prevent airborne transmission using various strategies such as oil sprinkling, air ionization, and air filtration.^62^ Evaluation of the effectiveness of each method is often limited due to the lack of a “gold-standard” virus and the standardized measurement approaches. A study by Létourneau et al. was aimed at identifying a set of viruses suitable for the evaluation of different mitigation strategies to prevent airborne transmission in swine shelters.^62^ As a result, two fecal viruses *Astrovirus group 2* and *Phage vB_AviM_AVP* of *Aerococcus* provided a working standard for testing mitigation strategies in animal shelters.^62^

Identification of the diversity and abundance of gut bacteriophages by meta-genomics is advancing public health through tracking fecal contaminations in water.^68,69^ Dutilh et al. identified *crAssphage*, a highly abundant bacteriophage in human fecal meta-genomes.^68^ The *crAssphage* protein sequences had no matches in the reference databases, highlighting the usefulness of nontargeted discovery approaches. Meta-genomic analysis of wastewater samples revealed pathogen contamination of aquatic environments and contributed to early detection and monitoring of pathogens of public health significance.

Other spread mitigation strategies focus on tracking the viral composition of wastewater and soil. There are debates over the microbiological safety of water reuse, and meta-genomics may provide evidence-based recommendations. Bacterial safety standards for the use of reclaimed water are well established, but the inclusion of viral monitoring would significantly complicate the task, as viruses respond differently to disinfectants and can infect at lower doses.^70^ Several studies have demonstrated the feasibility of meta-genomic analysis on wastewater to establish viral diversity and baselines for public health monitoring.^71–73^ A similar study on untreated sewage demonstrated the vast diversity of viruses, with only a small percentage of the viral genomes (∼11%) matching to known viruses.^72^ Standardization of protocols and the need for bioassays to assess viability and transmissibility of identified viruses were recognized as major challenges of meta-genomics wastewater monitoring.

## 4 Meta-Proteomics

### 4.1 Overview of Meta-Proteomics Platforms

Meta-proteomics has first appeared in the literature in 2004 and was defined as “the large-scale characterization of the entire protein components of environmental microbiota at a given point in time”.^74^ Meta-proteomics gained momentum in the last two decades due to significant improvements in mass spectrometry instrumentation and proteomics pipelines and their applications in basic research^75–80^ and translational studies.^81–91^

Translational and clinical meta-proteomics studies have emerged due to the need to analyze the composition of human microbiomes and their dynamics following bacterial and viral infections.^74,92^ A study re-examining meta-proteomes of human samples following SARS-CoV-2 infections identified bacterial coinfections that could impact the severity and outcomes of infections.^93^ The list of microbiomes examined using meta-proteomics is extensive and continues to grow, including the gut in healthy and disease states and the oral microbiome.^94–97^ In 2021, the meta-proteomic initiative was launched to promote dissemination and networking and foster further developments in the field.^98^ Meta-proteomics is still a relatively young field and would require further advances to ensure reliable and robust analysis of microbiomes. Bottom-up proteomic approaches by liquid chromatography and tandem mass spectrometry (LC-MS/MS) remain the main platform for meta-proteomics studies.^97,99^ The choice of a mass spectrometry platform for meta-proteomics studies should consider the study objectives, sample complexity, and sample amounts. For example, Orbitrap platforms with ultrahigh resolving power (100–500 K) and orthogonal peptide fragmentation modes would be beneficial for *de novo* sequencing of high amounts of proteins present in low-complexity samples. Time-of-flight (TOF) and trapped ion mobility spectrometry TOF (timsTOF) platforms with lower resolving power (40–60K) present highly competitive alternatives for fast identification of known proteins in low amounts of high-complexity samples, such as single-cell proteomic studies. Despite their low resolving power (1–5K), triple quadrupole and quadrupole-ion trap platforms would be competitive for rapid and highly multiplex quantification of known proteins in complex biological and environmental samples.^100–106^

### 4.2 Challenges of Meta-Proteomics Data Analysis and Interpretation

Challenges of meta-proteomics data analysis arise from the vast diversity of microorganisms and the large size of meta-proteomics databases used for peptide-spectrum matching.^60^ Computational, bioinformatic, and statistical challenges often make meta-proteomics inaccessible to researchers without specialized training in meta-omics.^107^ Specialized software, such as MetaLab,^107,108^ metaExpertPro,^109^ and ProteoStorm,^110^ were developed to facilitate meta-proteomics analysis.

False discovery rates (FDR) and false-negative identifications constitute the major challenges of meta-proteomics studies that rely on searching extremely large multispecies databases with millions of sequences.^111^ Common approaches for peptide identification are based on the probability that the experimental MS/MS spectra would match the predicted MS/MS spectra. The probability *P*-values of peptide-spectrum matches (PSM) are further adjusted for multiple comparisons, and expectation *e*-values are calculated for each peptide. Each PSM *e*-value is then compared to the global *e*-value cutoff that defines FDR at 1.0%, and PSMs with *e*-values higher than the global *e*-value are considered positive matches. FDR is typically calculated based on a target-decoy strategy: (i) all PSMs identified in the target proteome database constitute a sum of true-positive (TP) and false-positive (FP) identifications; (ii) all PSMs identified in the “decoy” database (a false database of the same size with all sequences reversed MATCH→HCTAM) constitute false-positive identifications. FDR is then calculated as FP/(TP+FP), and the *e*-value corresponding to FDR of 1.0% is set as the global cutoff. Such strategy serves well for peptide identification using the relatively small proteomes of single species, such as 20,436 canonical proteins of the UniProt human database. Searching the large multispecies databases shifts the global *e*-value cutoff to the smaller *e*-values in order to decrease the number of FP identifications. As a result, this shift leads to an increase in false-negative rates and missed identifications of true species.

Several two-stage database search strategies were proposed to mitigate the FDR challenge.^111–113^ Table 2 demonstrates the search of salivary proteomes collected at three different geographic locations, analyzed with similar mass spectrometry instruments and proteomic approaches, and reanalyzed with FragPipe software with a two-stage database search strategy.^113,114^ Identified bacterial species (38 to 49 species per data set) revealed ∼70% genera in common. Comparison with other previously reported studies revealed ∼75% common genera.^96^

**Table 2:** Two-Stage Meta-Proteomic Search of the Human Saliva Samples Collected at Three Different Geographic Locations

data set^a^	PXD043901	PXD035780	PXD050597	top 10 genera overall
**top 10 genera**	*Prevotella*	*Prevotella*	*Actinomyces*	*Prevotella*
	*Haemophilus*	*Neisseria*	*Streptococcus*	*Actinomyces*
	*Neisseria*	*Actinomyces*	*Prevotella*	*Neisseria*
	*Selenomonas*	*Veillonella*	*Veillonella*	*Veillonella*
	*Veillonella*	*Leptotrichia*	*Selenomonas*	*Selenomonas*
	*Fusobacterium*	*Fusobacterium*	*Rothia*	*Leptotrichia*
	*Leptotrichia*	*Porphyromonas*	*Leptotrichia*	*Streptococcus*
	*Actinomyces*	*Selenomonas*	*Neisseria*	*Haemophilus*
	*Campylobacter*	*Rothia*	*Alloprevotella*	*Fusobacterium*
	*Streptococcus*	*Haemophilus*	*Megasphaera*	*Rothia*
geographic region	Sweden	Greece	Canada	
total individuals	10	20	26	
total samples	30	40	26	
total file size, Gb	35	36	33	
gradient, min	85	50	135	
MS platform	Q Exactive HF	Q Exactive HF-X	Orbitrap fusion	
total proteins^b^	1277	673	635	
total species	49	45	38	
no. of genera in top 99% LFQ	25	22	17	

a Mass spectrometry data sets were downloaded from the PRIDE database (https://www.ebi.ac.uk/pride).

b The searches were completed by FragPipe software^113^ with MSBooster^115^ and a two-stage database search to ensure stage-specific FDR control.^113,116^ The human proteome (20,436 UniProt reviewed canonical proteins) was used in the first search, and all PSMs passing the 1% FDR level were removed. The remaining MS/MS spectra were searched against the Oral Microbiome database (919,221 proteins; www.hmpdacc.org/HMRGD) in the second search. Protein identifications were further filtered to keep identifications with at least one unique peptide peptide-spectrum match (PSM), at least 2 total PSM, >95% top peptide probability, and >95% protein probability. Each data set was searched individually, and the MaxQuant Label Free Quantitation (LFQ) algorithm was used for relative quantification.

## 5 Meta-Transcriptomics and Meta-Metabolomics

Other meta-omics techniques, such as meta-transcriptomics and meta-metabolomics, will further contribute to expansion of large-scale multiomics studies (Figure 3). Meta-transcriptomics typically relies on the reverse transcription of RNA transcript into cDNA and subsequent sequencing of cDNA with NGS platforms. Numerous applications of meta-transcriptomics in biomedical and environmental research were reported.^117^

**Figure 3:**
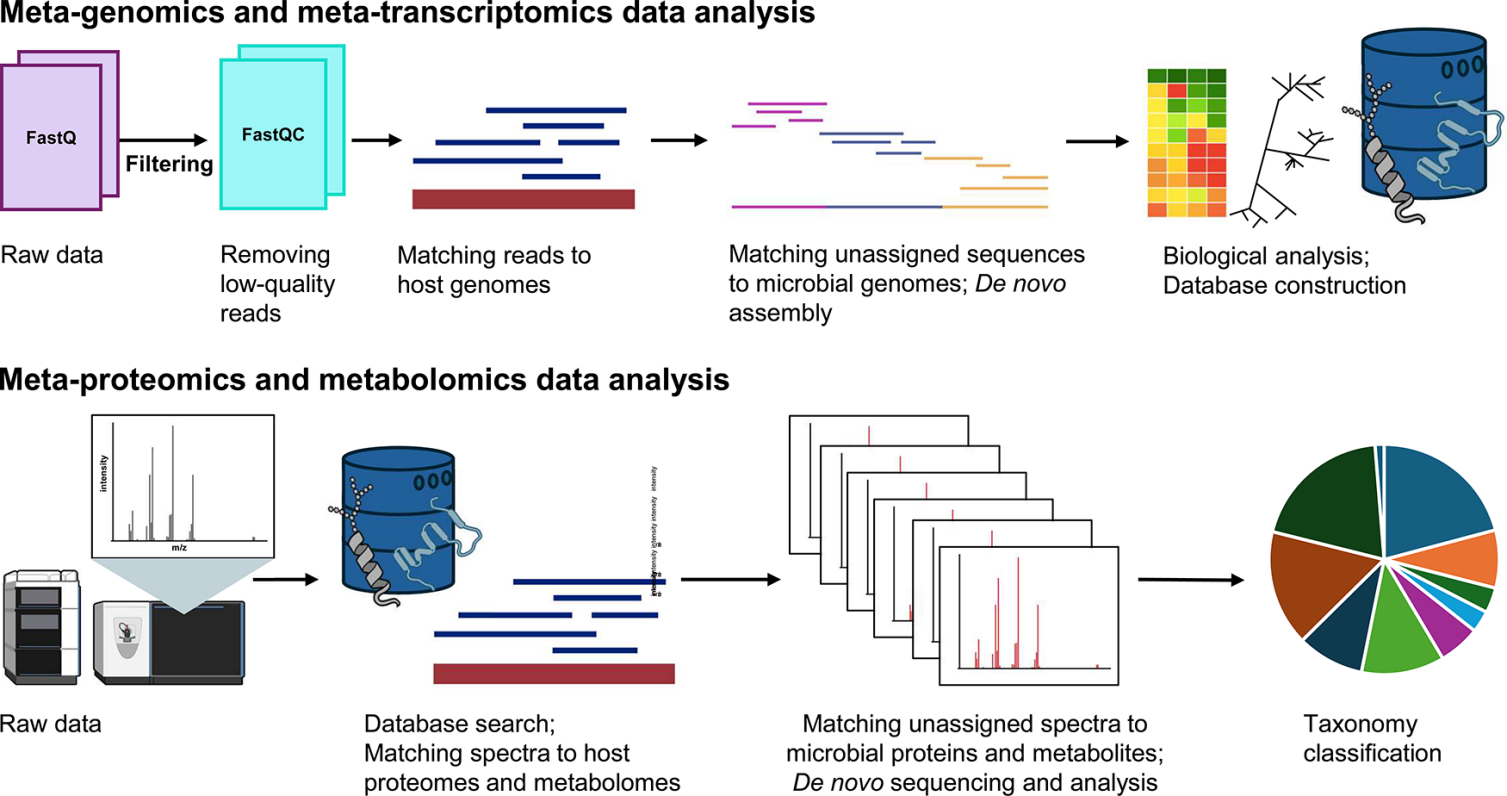
Meta-omics data analysis. Meta-genomics and meta-transcriptomics workflows (upper panel) process raw DNA and cDNA next-generation sequencing data over several steps such as removal of low-quality reads, matching the reads of acceptable quality to the high-abundance host genomes, and then matching unassigned sequences to the microbial sequence databases, or performing *de novo* assembly. The results are visualized as heatmaps with the abundance of microbial species and phylogenetic trees based on sequence similarity. Genomic and transcriptomic data are also utilized to construct host- and pathogen-specific proteome databases. Meta-proteomic and metabolomic workflows (lower panel) process raw mass spectrometry data over several steps such as feature detection (grouping isotopic patterns, detecting and recalibrating *m*/*z*, and detecting retention time and intensity of features), matching the identified features to peptide or metabolite databases, matching unassigned spectra to microbial peptide or metabolite databases, and calculating thresholds to filter out false positives. Proteomic workflows filter out false positives at three levels (peptide-spectrum matches, peptides, and proteins), and group peptides into proteins using the parsimony principle. The results of meta-proteomics analysis are visualized as heatmaps and taxonomic trees to provide the microbial composition of the sample.

Meta-metabolomics, or metabolomics, is aimed at identifying the diversity of metabolites within an entire ecosystem of an organism and its microbial communities. Metabolomics methods rely on untargeted mass spectrometry for global discovery and targeted mass spectrometry for validation or routing measurements. A recent multiomics study involving meta-metabolomics investigated longitudinal metabolic changes associated with aging and revealed the predominant nonlinear patterns, with the inflection points around 40 and 60 years.^118^ The various techniques and applications of metabolomics for microbiome studies and human health have been previously reviewed.^119–121^

## 6 Integration of Multiomics and Meta-Omics Studies

While meta-genomics is a powerful tool to identify and sequence microorganisms, it lacks information on dynamics of phenotypes and host–pathogen interactions, such as pathogenesis, transmissibility, and antibiotic resistance. Therefore, the identified genomes need to be annotated through functional assays and measurements of RNA transcripts, proteins, and metabolites, to determine the threat of microorganisms to the host and environment. Integration of multiomics into meta-omics studies (Figure 4) facilitates the discovery and verification of novel bacterial and viral species through improved transcript and protein annotations and insights into the protein function and metabolic activities.

**Figure 4:**
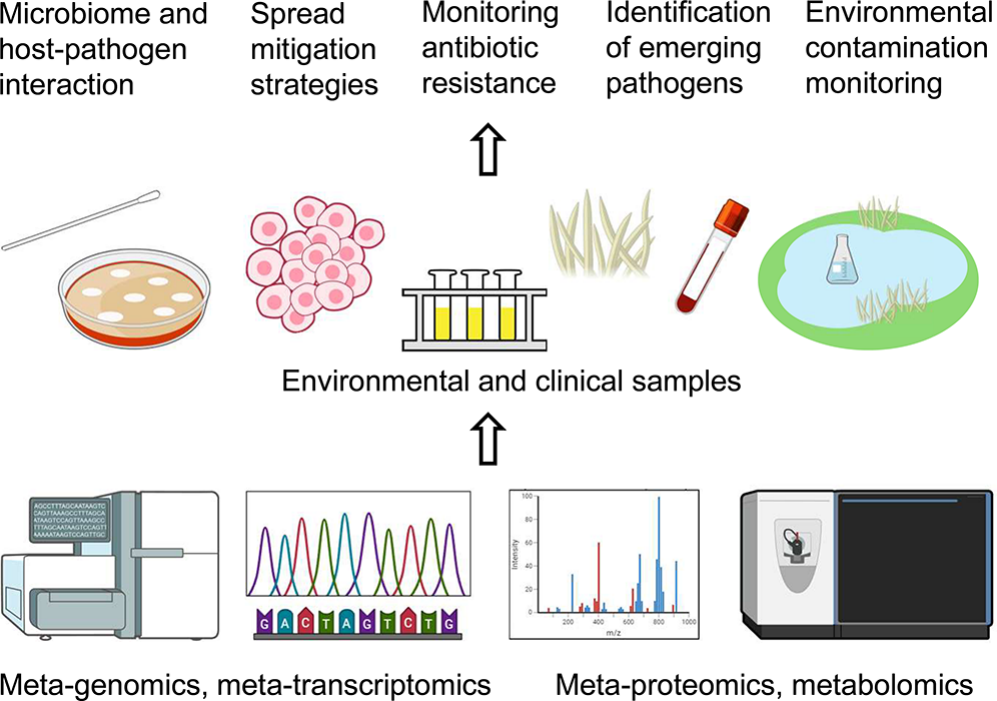
An overview of meta-omics applications. Meta-genomics and meta-transcriptomics utilize NGS to determine genomic sequences and RNA transcripts present in clinical specimens (tissues, blood, swabs) and environmental samples (water, soil, and air). Mass spectrometry is a principal platform for meta-proteomics and metabolomics. Combinations of multiomics and meta-omics techniques enable diverse approaches for clinical studies (host-pathogen interactions, analysis of microbiomes, and antibiotic resistance), public health safety (monitoring endemic and emerging pathogens), and environmental protection (monitoring impact of environmental contaminants).

### 6.1 Challenges of Multiomics Integration

Integration of multiomics data is an attractive idea, but it suffers from the often-vague meaning and implementation, with no single recipe widely accepted. Challenges of meta-omics integration reside in differences in complexity, dynamics, and biological functions at each -omics level.^122^ Thus, studies on the relationship between mRNA and protein levels revealed that only about 40% of the variance of protein levels could be explained by the levels of the corresponding mRNA.^123,124^ Furthermore, the studies of expression dynamics showed that mRNA fold changes at 5 h following stimulation correlated best with the protein fold changes after 12 h.^125,126^

A causative integration of -omics levels based on the “Central Dogma” of molecular biology (DNA→ RNA→ protein) represents the most useful approach for multiomics data integration.^127,128^ For example, homozygous nonsense mutations in the human *PAH* gene resulting in the truncated and nonfunctional forms of phenylalanine hydroxylase protein lead to substantially elevated levels of its substrate phenylalanine in blood serum, thus causing phenylketonuria, a metabolic disorder.^129^

If causative relationships based on the Central Dogma are not known, then correlative integration based on qualitative and quantitative changes at different -omics levels could be derived. However, correlative integration is challenging, may not be informative, and is prone to spurious correlations.^130–132^ While meta-omics provides an exciting approach for characterization of microorganism communities, there is a significant amount of data processing and filtering needed to generate new knowledge.^133,134^ Integrated meta-omics studies require dedicated strategies for sample collection and processing to preserve and extract not only nucleic acids but also proteins and metabolites from the same sample. Unlike nucleic acids, proteins and metabolites cannot be amplified and should be measured directly, thus resulting in additional preanalytical and analytical challenges of integrated meta-omics workflows.

Proper management of the vast volumes of data represents a technical challenge of multiomics and meta-omics integration strategies. Small-scale studies often rely on innovative lab-specific methods tailored to the study objectives, rather than routine and standardized pipelines. Analysis and independent reanalysis of large-scale omics data would require standardized methods for collection, cleaning, processing, interpretation, and data deposition to public repositories. Public repositories of omics data (Table 3) provide access to raw data, curate high-quality data, maintain databases, and facilitate *in silico* data mining and retrospective studies. Large-scale and high-quality data sets available in curated databases are an invaluable resource for machine-learning analysis, model training and validation, and exploration of complex multiparametric patterns.^135^ While individual -omics fields still have highly specialized workflows to analyze large-scale data, some recent initiatives (Omics Discovery Index^136^ and National Cancer Institute proteogenomics studies^137^) are leading the transformation toward generalized workflows and standardized strategies for large-scale multiomics studies.

**Table 3:** Public Repositories of -Omics Data^a^

data repository	curation	types of omics data	link
Omics Discovery Index	EBI-EMBL	28 omics repositories with programmatic access to data sets	www.omicsdi.org
GenBank	NCBI	genomics, transcriptomics	www.ncbi.nlm.nih.gov/genbank
European Nucleotide Archive	EBI-EMBL	genomics, transcriptomics, microbiomes	www.ebi.ac.uk/ena
National Cancer Institute Cancer Research Data Commons	NCI	genomics, transcriptomics, proteomics, imaging, epidemiology, clinical and translational data	https://pdc.cancer.gov/pdc
Genome Aggregation Database	Broad Institute	genomics (WGS, WXS)	https://gnomad.broadinstitute.org
The Accelerating Research in Genomic Oncology	International Cancer Genome Consortium	genomics (WGS, WXS), transcriptomics (RNA-Seq)	https://platform.icgc-argo.org
Dependency Map; Cancer Cell Line Encyclopedia	Broad Institute	genomics, transcriptomics, epigenetics, proteomics, and metabolomics (MS) data	https://depmap.org
PRIDE Database	EBI-EMBL	proteomics by MS	www.ebi.ac.uk/pride
MassIVE	UCSD	proteomics, metabolomics	https://massive.ucsd.edu
iProX	Beijing Proteome Research Center	proteomics by MS	www.iprox.cn
PeptideAtlas	Institute for Systems Biology	proteomics by MS	https://peptideatlas.org
Human Protein Atlas	Science for Life Laboratory	proteomics (antibody-based imaging), transcriptomics	www.proteinatlas.org
MetaboLights	EBI-EMBL	metabolomics	www.ebi.ac.uk/metabolights
Human Metabolome Database	University of Alberta	metabolomics (MS, GC-MS, and NMR data)	www.hmdb.ca
National Microbiome Data Collaborative	Lawrence Berkeley National Laboratory	microbiomes (meta-genomes, metatranscriptomes, meta-proteomes, metabolomes)	https://microbiomedata.org
MicrobiomeDB	NIH Bioinformatics Resource	microbiomes (16S rRNA seq)	https://microbiomedb.org

a Abbreviations: EBI-EMBL, European Bioinformatics Institute of the European Molecular Biology Laboratory; GC–MS, gas chromatography mass spectrometry; MS, mass spectrometry; NCBI, National Center for Biotechnology Information; NMR, nuclear magnetic resonance; NIH, National Institutes of Health; RNA-seq, RNA sequencing; UCSD, University of California San Diego; WGS, whole genome sequencing; WXS, whole exome sequencing.

### 6.2 Environmental Multiomics Studies

Multiomics and meta-omics approaches have been used to evaluate changes in the microbial communities following infections and environmental changes, examine the microbial resilience at water-treatment plants, and identify functional ecological niches.^138^ Meta-omics studies that revealed the genotype of microbes and further differentiated genetically similar organisms based on mRNA, proteins, and metabolites provided conclusions about the metabolic plasticity and niche complementarity of microbial communities. The combined -omics data allowed 94% of the identified proteins to be assigned to genes.^138^ Likewise, gas chromatography–mass spectrometry measurements linked 89% of identified metabolites to encoded enzymes.^138^ Integration of multiomics data for profiling of microbial community diversity lays the groundwork for applications of ecological engineering, host–microbiome studies, and their implications for animal and human health.

A multiomics study on swine H1N1, an influenza A subtype implicated in 2009–2010 Swine flu pandemic, revealed shifts to the functional composition and species abundance of the microbiome following infection.^139^ The multiomics approach monitored the development of the respiratory tract microbiome and characterized the gastrointestinal tract microbiome changes and immune response upon H1N1 infections. The combined meta-omics approach allowed for species identification based on the 16S RNA sequencing, protein expression levels, and metabolic changes. The composition and function of the gastrointestinal microbiome revealed similar changes of taxonomical composition across infected animals and found several groups of species that changed abundance at specific time points, providing a timeline to respond to infections. The results suggested that a similar meta-omics pipeline for fecal samples of flu-infected humans could facilitate noninvasive clinical diagnostics.

### 6.3 Perspectives of Multiomics in the Clinic

Clinical multiomics approaches promise to analyze large cohorts of human samples (Table 4) to enable improved diagnosis, informative profiling and monitoring of microbial communities, and prevention of infection outbreaks.^32^ The need for standardized protocols remains the major challenge for implementation of meta-genomics and meta-omics testing in clinical laboratories. Additional concerns include sample complexity, contamination, high background, computational power, and the need for intuitive and user-friendly software for straightforward data analysis and visualization.^107–110^ Various groups have proposed pipelines for “precision” meta-genomics featuring multiple “tracks” for samples based on time sensitivity, depth of analysis, and inclusion of data such as antimicrobial resistance.^147^ Retrospective meta-genomic analyses on clinical samples have been performed to demonstrate their utility and establish automated diagnostic pipelines.^148,149^ In a case study of cystic fibrosis, multiomics was used to monitor a patient for 2 years using meta-genomics, meta-transcriptomics, and metabolomics and enable a cystic fibrosis rapid response through monitoring the microbial changes during infections.^145^ A 2016 study demonstrated that the multiomics study implemented in ideal conditions (trained personnel working 24/7, available instruments, etc.) could provide useful clinical information within 48 h.^146^ The need for individualized baselines presents issues of routine sample collection and data analysis but could be relatively easily established for patients with chronic diseases.^145^ Overall, clinical genomics initiatives laid out a well-established foundation to implement meta-genomic pipelines in clinical laboratories. However, further work is needed to set guidelines and standards before the full clinical utility of meta-genomics and meta-omics can be established.

**Table 4:** Recent Clinical Meta-Omics Studies, Sample Types, and Major Findings

study type	sample numbers	platform	major findings	source
meta-genomics	81 sputum samples	NGS: MinION nanopore	identified pathogens and antibiotic resistance	^140^
meta-genomics	24 spiked blood samples	NGS: MinION nanopore	identified bacterial species in blood and detected antibiotic resistance	^141^
meta-genomics	40 wound samples	NGS: MinION nanopore	identified four high-priority pathogens and antimicrobial resistance genes	^142^
meta-genomics	1120 bovine fecal samples	NGS: NovaSeq	identified a total of 110 viruses and 1011 bacterial genera	^41^
meta-genomics	17 vitreous specimens	NGS: Ion Torrent	pathogens in 94% of nonculturable samples (PCR rate was only 69%)	^143^
meta-genomics	2 natural whey starter cultures	NGS: MiSeq, Sequel, MinION nanopore	obtained complete *de novo* sequences of dominant bacteria in cultures	^44^
meta-genomics	3 oral cavity samples	NGS: Genome Analyzer IIX	identified 135 genera, with some taxa never before seen in oral cavity	^49^
meta-genomics	100 FFPE tissue blocks	NGS: NextSeq	identified the presence of taxa associated with colorectal cancer	^144^
meta-genomics	9 bat specimens	NGS: HiSeq	determined the virome of bat species, including new mammalian viruses	^66^
meta-genomics	18 air in swine shelters	NGS: MiSeq	identified eight phages and chose two phages as viral proxies for monitoring	^62^
meta-genomics	86 and 12 fecal meta-genomes	NGS: Public repository data	identified an abundant bacteriophage *crAssphage* suitable as a marker of fecal contamination of water.	^68,69^
meta-genomics	2 untreated sewage samples	NGS: Genome Analyzer IIX	reported 89% of assembled reads unmatched to known viruses	^72^
meta-genomics	340 human fecal samples	NGS: NovaSeq	meta-genomics and meta-proteomics identified nutritional exposure and species-specific functional dysbiosis in inflammatory bowel disease patients	^92^
meta-transcriptomics, meta-proteomics, meta-proteomics		MS: Q Exactive HF-X		
meta-proteomics	18 fecal samples	MS: Q Exactive HF Orbitrap	identified 96 protein markers of gut microbiome response to arsenic	^95^
meta-proteomics	34 saliva samples	MS: Orbitrap Q Exactive Plus	identified oral meta-proteome a lung cancer-associated bacteria	^96^
meta-proteomics	518 gut aspirate samples	MS: Orbitrap Exploris 480	identified changes in proteome-level functional redundancy during exposure to xenobiotics and gut inflammation	^99^
transcriptomics, proteomics	blood, stool, nasal samples of 108 participants	NGS: HiSeq	identified nonlinear patterns in molecular markers of aging tracked over several years	^118^
metabolomics		MS: TripleTOF 6600, Q Exactive Plus		
meta-genomics	biomass of 53 floating sludge islets	NGS: Genome Analyzer IIX	revealed phenotypic plasticity and niche complementarity in oleaginous microbial populations from a biological wastewater treatment plant	^138^
meta-transcriptomics				
meta-proteomics		MS: Orbitrap Elite, 5975C GC-MS		
metabolomics				
meta-transcriptomics, meta-proteomics, metabolomics	68 fecal samples	NGS: MiSeq	established a microbiome baseline and changes upon H1N1 influenza infection in swine	^139^
		MS: Orbitrap Velos		
		NMR: Advance II		
meta-genomics	serial sputum samples	NGS: Genome Analyzer IIX	identified *E. coli* shiga toxin as a factor of complications in cystic fibrosis	^145^
meta-transcriptomics				
metabolomics		MS: Maxis qTOF		
meta-genomics	7 participants (skin, feces, oral, swabs)	NGS: MiSeq	presented an integrated omics pipeline for the analysis of human and environmental samples in under 48 h	^146^
metabolomics		MS: Q Exactive		

## 7 Multiomics Health Initiatives

The emergence of technologies for in-depth analysis of biological molecules in human cells, tissues, and fluids motivated the establishment of integrated multiomics health initiatives such as P4 Medicine and One Health. The “100-person wellness project” was among the first initiatives and tracked over 100 individuals using genomic, metabolomic, and microbiome tests performed every 3 months in addition to exercises and sleep monitoring.^150^ However, the study results raised skepticism whether such extensive monitoring was required. Despite clear promises and technological advantages, the early initiatives utilizing conventional and convenient workflows for multiomics and meta-omics data acquisition and analysis revealed challenges and serious issues, including false positives, data overfitting, and overtesting (Figure 5). The study outcomes emphasized the need for strict guidelines, standardization, and specialized personnel training.

**Figure 5:**
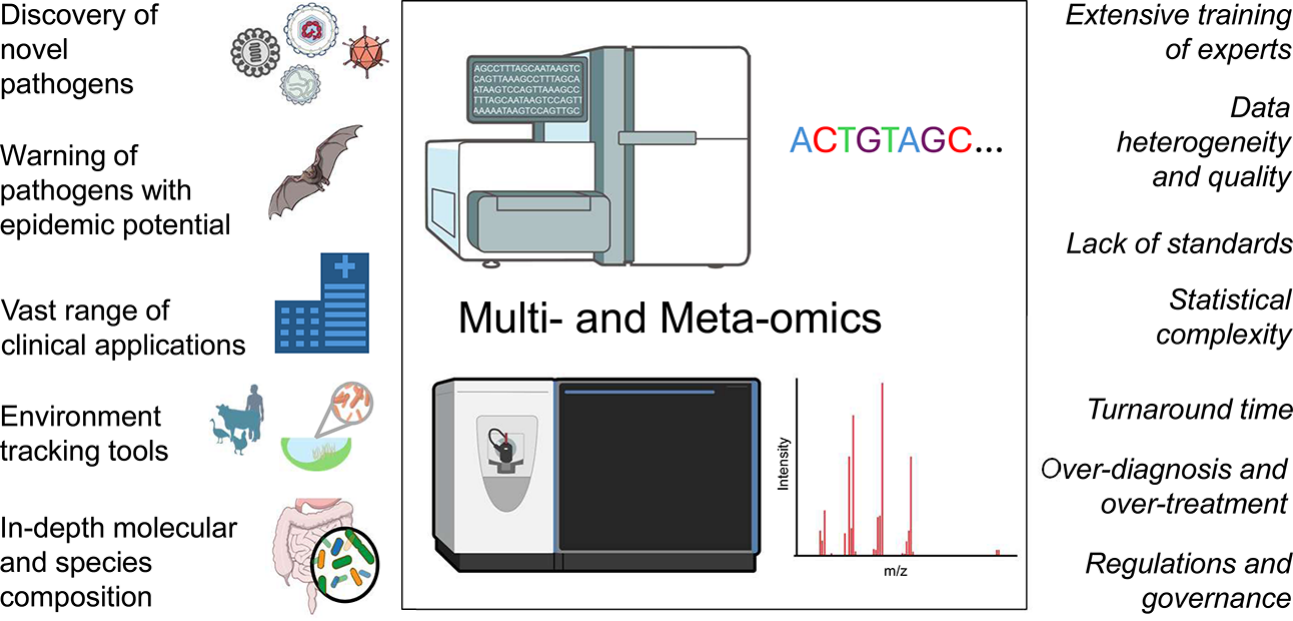
Advantages and challenges of multiomics and meta-omics studies. *Advantages* (left panel): Discovery of novel pathogens and warning of emerging zoonotic viruses will tremendously impact public health. First, it will allow for better diagnostic medicine as more pathogens are characterized. Second, early identification of zoonotic pathogens with epidemic potential will help to predict, prepare, and prevent widespread transmission of infections, reducing the strain on healthcare systems. The meta-omics sample range (environmental, agricultural, or clinical) enables many applications, making meta-omics an attractive field. Lastly, the depth of knowledge obtained from meta-omic and multiomic analyses (genes, mRNA, proteins, and metabolites) will improve our understanding of host–pathogen interactions. *Challenges* (right panel): Meta-omics and multiomics experts require extensive training, increasing the cost and exclusivity of such tests. Meta-omics and muti-omics analysis can lead to overdiagnosis and overtreatment of patients, unnecessarily straining healthcare systems. A significant challenge in meta-omics research is cross-lab standardization, especially considering the array of sample types used. The meta-omics pipeline is currently labor and time-intensive, limiting its real-world usefulness, though efforts for automated pipelines are under way. Finally, collaborations of local, regional, national, and international governments will be required to implement meta-omics and multiomics empowered One Health initiatives.

The P4 Medicine (Predictive, Preventative, Personalized, and Participatory) has emerged with a central goal to tackle the challenges of slow-progressing chronic illnesses, the major burden on healthcare systems, and focused on early detection and interventions to prevent chronic diseases and delay their progression.^151^ The Predictive approach to identify and eliminate the risk factors and discover predictive biomarkers was essential to enable disease prevention.^151,152^ The Preventative approach consisted of age- or sex-specific programs, such as vaccination strategies targeting *Human papillomavirus* and *Helicobacter pylori* to prevent human cancers of viral and bacterial origin.^153,154^ The Personalized approach referred to drastically different responses to different treatments among the patients, while the Participatory approach suggested the patient’s responsibility for optimizations of their health. P4 Medicine revealed the issues of overtesting, overdiagnosis, overtreatment, and overcharging of patients.^155^ Overtesting results in false positives from otherwise unnecessary tests; overdiagnosis is diagnosing a disease that would not become clinically significant or pose a threat, discomfort, and changes in quality of life; overtreatment is an unnecessary treatment with no benefits to the patient. Finally, unnecessary tests and treatments burden the healthcare systems through overcharging and health inequity. While P4 Medicine presents an idealistic solution to tackle chronic diseases, its realistic implementations are challenging.

A recently emerged “One Health” initiative was aimed at another potentially idealistic approach to maintain the health of humans, animals, and the environment through the numerous interdisciplinary and collaborative initiatives.^156^ The key concept of One Health is a collaboration between individuals, Public Health, local nongovernmental organizations, governments, and international intergovernmental organizations. One Health considers zoonosis-triggered epidemics and pandemics, in addition to human, animal, and environmental health threats such as antimicrobial resistance and pollution.^157^ Most importantly, the One Health initiative stresses the importance of the human–animal–environment interface as a source of emerging diseases. Following the outbreak of COVID-19 pandemic, several groups called for the implementation of One Health approach as a solution to combat and prevent future pandemics.^158–160^ Additionally, in the context of antimicrobial resistance, One Health examined the impact of introducing antibiotic-resistant clones on local and global environmental microbiomes and the human- and animal-associated microbiomes.^161^ The main drivers of resistance were the exhaustive use of antibiotics in clinics and livestock farms, as much as two-thirds of the total antibiotic use.^161,162^ Additional drivers included a lack of infrastructure (such as proper sewage disposal) to inhibit the spread of resistant clones, as well as climate change that increased human-zoonotic interactions. A prominent example of cross-border and antibiotic resistant infections was an outbreak of New Delhi metallo-beta-lactamase-producing carbapenem-resistant *Enterobacteriaceae* in Italy in 2018.^163^

One Health initiatives are not without criticism and barriers to implementation.^157^ The ecological, environmental, and social aspects of One Health are often overlooked for biochemical aspects of diseases and thus hinder the societal implementation of One Health initiatives. Additionally, health divisions of local and national governments, as well as international health agencies, need to be involved in the initiatives for their successful implementations.^164^

## 8 Future Outlook and Conclusions

Microbes are diverse, ubiquitous, and highly integrated into ecosystems and have a vast impact on human and animal health. Conventional and targeted methods for pathogen identification, such as PCR, are efficient at detection but ignore the diversity of pathogens in the environmental and clinical samples. Meta-genomics approaches are emerging as a powerful toolbox to discover and monitor evolving microbial communities, respond to novel pathogen outbreaks, and track environmental contamination. The valuable end goals multiomics and meta-omics studies are their clinical implementations to improve diagnostic and therapeutic approaches. Integrated multiomics methods can reveal the detailed composition and diversity of genotypes and functional phenotypes of microbial communities. Multiomics approaches are not without challenges such as the need for highly qualified personnel, specialized equipment, complex data analysis, lack of standardization, and long turnaround time. As pandemic preparedness in a post-COVID-19 world moves to the forefront of scientific research, knowledge of diversity and depth of microbial communities and identification of their pathogenic potential will help to predict, respond, and prevent future threats to public health.
